# The Baseline and Longitudinal Changes of PCC Connectivity in Mild Cognitive Impairment: A Combined Structure and Resting-State fMRI Study

**DOI:** 10.1371/journal.pone.0036838

**Published:** 2012-05-18

**Authors:** Zhiqun Wang, Peipeng Liang, Xiuqin Jia, Guangwei Jin, Haiqing Song, Ying Han, Jie Lu, Kuncheng Li

**Affiliations:** 1 Department of Radiology, Xuanwu Hospital of Capital Medical University, Beijing, China; 2 Department of Neurology, Xuanwu Hospital of Capital Medical University, Beijing, China; 3 Key Laboratory for Neurodegenerative Diseases, Ministry of Education, Beijing, China; Institution of Automation, CAS, China

## Abstract

The baseline and longitudinal changes of the posterior cingulate cortex (PCC) connectivity were assessed in order to clarify the neural mechanism of mild cognitive impairment (MCI). Twenty-eight right-handed subjects (14 MCI patients and 14 healthy elders) participated in this study. Clinical and neuropsychological examinations were performed on all the subjects. PCC functional connectivity was studied by examining the correlation between low frequency fMRI signal fluctuations in the PCC and those in all the other brain regions. Additionally, we traced all the MCI patients and compared their PCC connectivity in the initial stage and that in 3 years later. We also explored the relationship between the PCC functional connectivity strength and cognitive performances. Our results are as follows: Functional connectivity between the PCC and a set of regions is decreased in MCI patients. Most of these regions are within the default mode network (DMN). Three years later, the regions of superior frontal gyrus (SFG) and middle frontal gyrus (MFG) presented further decreased connectivity to the PCC in MCI. In addition, we also find enhanced functional connectivity between PCC and medial prefrontal cortex (MPFC), PCC and anterior cingulate cortex (ACC) in MCI patients. At last, our research also shows that the PCC connectivity with some regions significantly correlates with the cognitive performances of patients as measured by mini-mental state examination (MMSE), and California verbal learning test (CVLT) scores. The baseline and longitudinal changes of the PCC connectivity in our study suggest that impairment and compensation coexist in the disease progress of MCI patients.

## Introduction

Alzheimer’s disease (AD) is the most prevalent form of dementia worldwide. The neuropathological changes of AD are characterized by amyloid-β plaques, neurofibrillary tangles and neuronal loss [1]. Mild cognitive impairment (MCI) refers to the most important at-risk state of AD. It has a high probability of evolving toward AD at a rate of 10–15% per year [2]. The pathogenesis of AD and MCI, however, remains rather unclear.

In the past several years, many neuroimaging studies, including structural MRI and functional MRI (fMRI), have been done to try to find out the pathogenesis of AD and MCI. Recently, resting-state functional connectivity has raised increasing attention which studied the correlation in low frequency fluctuations (LFF) (<0.1 Hz) of the blood oxygen level-dependent (BOLD) signal. It has been used to investigate the strength of distributed networks in the brain of AD and MCI by exploring the functional connection of spatially distinct but temporally correlated regions [3]–[18].

Several resting-state fMRI studies have investigated the neuronal integrity in the brain of the AD patients with different methods. By using regions of interest (ROI)-based functional connectivity approaches, the researchers found reduced functional integrity related to hippocampus [3], [4], prefrontal regions [5] and posterior cingulate cortex (PCC) [6] in AD. Using independent component analysis (ICA), Greicius and his colleagues [7] showed AD-related reduction of spontaneous brain activity within a default-mode network (DMN) including the PCC and medial prefrontal cortex (MPFC).

To our knowledge, only a few resting-state fMRI studies were applied in MCI patients. Liang et al. [8] explored the integrity of task-positive networks in MCI patients. More researchers focused on the changes of task negative networks such as DMN in MCI patients. However, those results were not very consistent. Using PCC connectivity, Bai et al. [9] found the disruption of PCC–temporal connectivity in the MCI patients. Using ICA, Sorg et al. [10] found the DMN regions and executive attention network had markedly reduced LFFs activity in the MCI patients. Qi et al. [11] found that MCI patients exhibited decreased functional activity in the DMN regions, while increased activity in the left prefrontal cortex and other parietal, temporal regions. Another study found no changes of DMN in MCI patients [12].

As stated above, only a few studies investigated the DMN changes in MCI patients, and there was some discrepancy in the previous studies. Most importantly, we are still unclear how the DMN changes with the cognitive decline,for the longitudinal study of resting fMRI in MCI is extremely limited and there remains the urgency to try to identify the patterns of altered function in MCI.

PCC is commonly identified as a critical node in the DMN. In the current study, we used PCC as the ROI to explore its altered connections with the other brain regions. We also examined the gray matter atrophy in MCI patients and used it as covariate to analyze the resting functional connectivity. We hypothesized that the resting-state PCC connectivity with some brain regions would be altered in the MCI patients, with the decline of cognition.

## Materials and Methods

### Subjects

This study was approved by the Medical Research Ethics Committee of Xuanwu Hospital. Thirty-two right-handed subjects (16 MCI patients and 16 healthy elders) participated in this study. The MCI subjects were recruited from patients who had consulted a memory clinic for memory problems at Xuanwu Hospital, Beijing, China. The healthy elderly controls were recruited from the local community through advertisements. Written informed consent was obtained from each participant. Some participants may have the reduced capacity to understand, for example, they may not understand the informed consent document to the full extend due to their age. In this case, their guardians consented on behalf of them.

Participants with MCI had memory impairment but did not meet the criteria for dementia. The criteria for identification and classification of subjects with MCI [19] was: (a) impaired memory performance on a normalized objective verbal memory test; (b) recent history of symptomatic worsening in memory; (c) normal or near-normal performance on global cognitive tests [Mini-Mental State Examination (MMSE) score >24], as well as on activities of daily living scale; (d) global rating of 0.5 on the clinical dementia rate (CDR) Scale, with a score of at least 0.5 on the memory domain; (e) absence of dementia.

The criteria for healthy elderly are as follows: (a′) no neurological or psychiatric disorders such as stroke, depression, epilepsy; (b′) no neurological deficiencies such as visual or hearing loss; (c′) no abnormal findings such as infarction or focal lesion in conventional brain MR imaging; (d) no cognitive complaints; (e′) MMSE score of 28 or higher; (f′) CDR score of 0.

The exclusion criteria are as follows: all subjects with contra-indications to MRI such as pacemaker, cardiac defibrillator, implanted material with electric or magnetic system, vascular clips or mechanical heart valve, cochlear implant or claustrophobia will be excluded; stroke, psychiatric diseases, drug abuse, moderate to serious hypertension, and systematic diseases are ruled out; intellectual disability can also not be included in the group.

Data from four subjects (2 MCI patients and 2 healthy elders) were excluded due to excessive motion (see Image preprocessing). Clinical and demographic data for the remaining 28 participants were shown in Table 1a.

**Table 1:** Demographics and clinical finding in MCI subjects and controls.

a	MCI(*N* = 14)	Controls(*N* = 14)	*p* value
Sex, female/male	8/6	8/6	>0.99#
Age, year	69.64±6.88	68.07±7.46	0.583*
Education, year	11.00±3.59	9.14±3.66	0.082*
MMSE	26.64±1.01	28.57±0.65	<0.05*
CVLT(immediate)	7.51±0.85	10.84±0.57	<0.05*
CVLT(short time)	6.76±0.48	11.16±0.54	<0.05*
CVLT(long-time)	5.47±0.76	11.60±0.58	<0.05*
CDT	5.54±0.52	9.65±0.45	<0.05*
**b**	**MCI1(** ***N*** ** = 7)**	**MCI2(** ***N*** ** = 7)**	***p*** ** value**
Sex, female/male	5/2	–	–
Age, year	70.85±3.57	–	–
Education, year	10.14±4.05	–	–
MMSE	26.57±1.27	25.01±1.94	>0.05
CVLT(immediate)	7.68±1.56	7.00±1.84	>0.05
CVLT(short time)	9.00±2.94	8.14±2.05	>0.05
CVLT(long-time)	5.86±1.67	4.85±1.41	>0.05
CDT	5.71±1.25	4.71±1.21	>0.05
**c**	**control 1(** ***N*** ** = 9)**	**control2(** ***N*** ** = 9)**	***p*** ** value**
Sex, female/male	5/4	–	–
Age, year	67.85±3.57	–	–
Education, year	9.14±3.12	–	–
MMSE	28.65±1.23	28.76±1.04	>0.05
CVLT(immediate)	11.56±1.25	10.96±1.34	>0.05
CVLT(short time)	11.87±1.34	11.62±1.12	>0.05
CVLT(long-time)	11.56±1.37	11.25±1.45	>0.05
CDT	8.74±1.05	8.21±1.21	>0.05

MMSE, Mini-Mental State Examination; values are means ± SD. WHO-UCLA CVLT, California verbal learning test; immediate, immediate recall of learning verbal; short-time, short time delayed free recall; long-time, long time delayed free recall; CDT, clock drawing test.

# The p value was obtained using a Pearson x^2^ two-tailed test, with continuity correction for n<5.

* The p value was obtained by a two-sample two-tailed t test.

We traced 9 MCI patients and data from 2 out of those 9 subjects were excluded due to excessive motion. Clinical and demographic data for the remaining 7 patients were shown in Table 1b. Here we take the initial stage of 7 MCI patients as MCI1 group and stage of the same MCI patients 3 years later as MCI2 group.

We traced 9 controls in 3 years. Clinical and demographic data for the 9 controls were shown in Table 1c. Here we take the initial stage of 9 controls as control1 group and stage of the same control patients 3 years later as control2 group.

### Data Acquisition

MRI data acquisition was performed on a SIEMENS Trio 3-Tesla scanner (Siemens, Erlangen, Germany). Foam padding and headphones were used to limit head motion and reduce scanner noise. The subjects were instructed to hold still, keep their eyes closed and think nothing in particular. Functional images were collected axially by using an echo-planar imaging (EPI) sequence [repetition time (TR)/echo time (TE)/flip angle (FA)/field of view (FOV) = 2000 ms/40 ms/90°/24 cm, resolution = 64×64 matrix, slices = 28, thickness = 4 mm, gap = 1 mm, bandwidth = 2232 Hz/pixel]. The scan lasted for 478 s. 3D T1-weighted magnetization-prepared rapid gradient echo (MPRAGE) sagittal images were collected by using the following parameters: TR/TE/inversion time (TI)/FA = 1900 ms/2.2 ms/900 ms/9°, resolution = 256×256 matrix, slices = 176, thickness = 1.0 mm.

**Figure 1:**
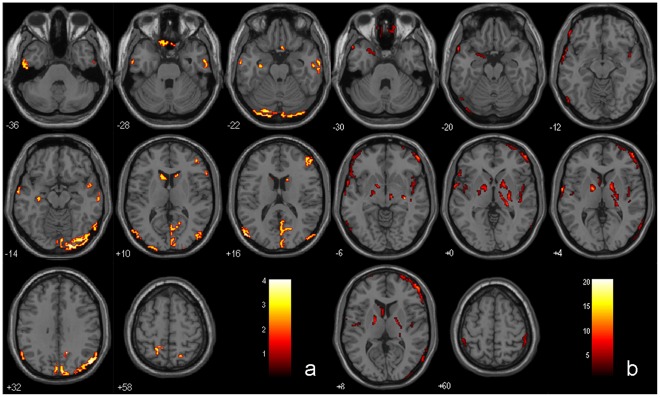
Gray matter intensity maps in MCI group using VBM analysis. a) Brain regions showing gray matter atrophy in MCI group comparing to control group. b) Brain regions showing gray matter atrophy in MCI2 group comparing to MCI1 group.

**Table 2:** Regions of gray matter atrophy in MCI subjects comparing to controls.

Regions NC vs. MCI	BA	Cluster size	Coordinates (MNI)			T-score
			x	y	z	
Lt. Middle TemporalGyrus	19	222	−52	−80	16	4.05
Lt. Fusiform Gyrus	18	152	−20	−94	−22	3.78
Lt. Middle TemporalGyrus	21	255	−64	−4	−14	3.41
Rt. Inferior TemporalGyrus	21	159	66	−8	−22	3.41
Rt. Inferior TemporalGyrus	37	88	64	−60	−8	3.15
Rt. ParahippocampalGyrus	34	47	−32	−16	−20	3.09
Rt. Superior TemporalGyrus	38	37	48	2	−14	2.86
Rt. Inferior FrontalGyrus	46	115	48	44	16	3.51
Lt. Superior FrontalGyrus	6	55	−14	−4	74	3.44
Rt. Rectal Gyrus	11	37	4	16	−24	3.33
Lt. Rectal Gyrus	11	65	−4	22	−30	3.29
Rt. Inferior FrontalGyrus	45	67	62	26	4	3.01
Rt. Precuneus	31	1209	14	−58	30	4.14
Lt. Precuneus	7	66	−18	−52	58	3.43
Lt. Inferior ParietalLobule	40	80	−60	−50	42	3.18
Rt. Superior ParietalLobule	7	51	24	−68	54	3.03
Rt. Inferior OccipitalGyrus	17	710	24	−98	−18	3.89
Lt. Middle OccipitalGyrus	18	158	−32	−100	0	3.66
Rt. Lingual Gyrus	18	34	18	−56	2	3.14
Lt. Caudate	–	91	−12	22	6	3.11
Rt. Caudate	–	40	14	22	10	2.76

### Image Preprocessing

All analyses were conducted using statistical parametric mapping (SPM5) package (http://www.fil.ion.ucl.ac.uk/spm). The first 10 volumes of the functional images were discarded for the signal equilibrium and participants’ adaptation to the scanning noise. The remaining 229 fMRI images were first corrected for within-scan acquisition time differences between slices and then realigned to the first volume to correct for interscan head motions. None of the participants had head motion of more than 1.5 mm maximum displacement in any of the *x*, *y*, or *z* directions, or 1.5° of any angular motion throughout the whole course of scan. Next, we spatially normalized the realigned images to the standard echo-planar imaging template and resampled them to 3×3×3 mm^3^. Subsequently, the functional images were spatially smoothed with a Gaussian kernel of 4×4×4 mm^3^ full width at half maximum (FWHM) to decrease spatial noise. Following this, temporal filtering (0.01 Hz<*f*<0.08 Hz) was applied to the time series of each voxel to reduce the effect of low-frequency drifts and high-frequency noise [20]–[22] by using Resting-State fMRI Data Analysis Toolkit (http://resting-fmri.sourceforge.net). To further reduce the effects of confounding factors, we also used a linear regression process to further remove the effects of head motion and other possible sources of artifacts [23]: (1) six motion parameters, (2) whole-brain signal averaged over the entire brain, (3) linear drift, (4) the white matter signal, (5) the cerebral spinal fluid (CSF) signal.

**Table 3:** Regions of gray matter atrophy in MCI2 comparing to MCI1 subjects.

Regions MCI1 vs. MCI2	BA	Cluster size	Coordinates (MNI)			T-score
			x	y	z	
Lt. Superior TemporalGyrus	22	191	−66	−2	4	14.76
Lt. Fusiform Gyrus	18	50	−26	−94	−26	9.36
Rt. Insula	13	192	42	−20	−8	7.63
Lt. ParahippocampalGyrus	34	84	−14	0	−18	6.70
Rt. Superior TemporalGyrus	22	81	46	2	−12	6.04
Lt. Superior TemporalGyrus	22	51	−48	2	−2	4.73
Lt. Insula	13	51	−44	−10	10	4.40
Rt. Middle Frontal Gyrus	46	1343	54	50	14	21.49
Rt. Superior Frontal Gyrus	6	60	−8	0	78	13.72
Rt. Orbital Gyrus	11	100	20	44	−28	11.88
Lt. Middle Frontal Gyrus	46	684	−46	46	30	11.69
Lt. Orbital Gyrus	11	38	−8	38	−34	9.25
Rt. Precentral Gyrus	6	49	44	−4	38	9.11
Lt. Superior Frontal Gyrus	11	34	−18	52	−28	8.24
Lt. Inferior Frontal Gyrus	47	167	−56	20	−2	7.73
Rt. Paracentral Lobule	5	36	20	−46	54	14.50
Rt. Postcentral Gyrus	3	167	18	−14	82	7.43
Rt. Inferior Parietal Lobule	40	39	52	−40	60	5.95
Lt. Inferior Parietal Lobule	40	38	−48	−38	54	5.29
Rt. Precuneus	7	36	16	−50	46	3.63
Rt. Middle Occipital Gyrus	19	572	44	−92	14	10.12
Lt. Middle Occipital Gyrus	19	65	−56	−72	−10	8.53
Rt. Lentiform Nucleus	–	222	30	−20	−4	24.79
Lt. Lentiform Nucleus	–	133	−16	−2	4	13.57
Rt. Subthalamic Nucleus	–	95	10	−16	−6	12.96
Lt. Caudate	–	104	−6	0	14	8.63
Lt. Lentiform Nucleus	–	40	−18	−10	−6	7.04

### Definition of Seed Regions

PCC seed region was 6-mm-diameter spheres centered on previously published foci (Talairach coordinate: −5 −49 40) [23], which has been widely used in many previous studies. The BOLD time series of the voxels within the seed region was averaged to generate the reference time series.

### Functional Connectivity Analysis

A correlation map was produced for each subject by computing the correlation coefficients between the reference time series and the time series from all the other brain voxels. Correlation coefficients were then converted to *z* values using Fisher’s *r*-to-*z* transform to improve the normality [22].

### Voxel Based Morphometry (VBM) Analysis

A VBM analysis of structural images was performed to control for the possible confounding effect of atrophy on the functional results [14], [24]. Gray matter intensity maps were obtained by the unified segmentation algorithm [25] as described in the Data Preprocessing section. Then, gray matter intensity maps were spatially smoothed with a Gaussian kernel of 10 mm FWHM. Finally, a two-sample t-test was performed on the smoothed gray matter intensity maps to examine the regional gray matter atrophy in MCI patients as compared to healthy controls. The statistical threshold was set at P<0.001 and cluster size >324 mm^3^, which corresponded to a corrected P<0.05 (using the AlphaSim program with parameters: FWHM = 10 mm, within the group gray matter mask).

**Table 4:** Regions of gray matter atrophy in control2 subjects comparing to control1 subjects.

Regions control2 vs.control1	BA	Cluster size	Coordinates(MNI)				T-score	
			x	y		z		
Lt. Middle Temporal Gyrus	21	160	−60	−21	−6		8.66	
Rt. Middle Temporal Gyrus	21	80	63	−6	−15		7.75	
Rt. Middle Temporal Gyrus	21	11	54	−33	0		5.19	
Rt. Middle Frontal Gyrus	8	543	21	27	45		9.23	
Lt. Middle Frontal Gyrus	8	521	−21	24	45		9.07	
Lt. Precuneus	31	393	−6	−51	33		24.44	
Rt. Precuneus	31	383	9	−63	27		10.90	
Lt. Inferior Parietal Lobule	40	189	−51	−63	39		8.51	
Rt. Supramarginal Gyrus	40	87	54	−54	33		8.06	
Lt. Caudate	–	26	−12	15	12		5.98	

### Statistical Analysis

The individual z value was entered into a random effect one-sample t-test to determine the brain regions showing significant connectivity to the PCC within each group under a combined threshold of P<0.01 and cluster size ≧ 405 mm^3^. This yielded a corrected threshold of P<0.001, determined by Monte Carlo simulation using the AlphaSim program with parameters: FWHM = 4 mm, within the gray matter mask. This procedure produced significant PCC functional connectivity t-statistic maps in four groups (14 MCI patients, 14 healthy controls, MCI1 and MCI2). We then made two masks by combining the corresponding two t-statistic maps (i.e., 14 MCI patients and 14 healthy controls; MCI1 and MCI2), and then used them respectively for analyzing the corresponding group differences.

The *z* values were also entered into a random effect two-sample *t*-test to identify the regions showing significant differences in connectivity to PCC between 14 MCI patients and 14 healthy controls, and between MCI1 and MCI2. Voxels survived a corrected threshold of P<0.05 (group differences between 14 MCI patients and 14 healthy controls: single voxel threshold of P<0.01 and cluster size 216 mm^3^, using the AlphaSim program with parameters: FWHM = 4 mm, with mask; group differences between MCI1 and MCI2: single voxel threshold of P<0.05 and cluster size 270 mm^3^, using the AlphaSim program with parameters: FWHM = 4 mm, with mask) were considered to show significant difference between the two groups.

### Correlation Analysis of PCC Connectivity and Neuropsychological Measures

In order to explore whether PCC connectivity varies with the disease progression and memory performances in MCI patients, a further correlation analysis between the PCC connectivity and neuropsychological performances was done. First, averaged z-values of each cluster with significant group differences were extracted. Then, Pearson’s correlative analysis were performed to examine the possible relationships between the z-values and neuropsychological performances [California verbal learning test (CVLT): Immediate Recall, Short Delayed Recall, Long Delayed Recall, and MMSE] in MCI patients.

**Table 5:** Regions of decreased connectivity to the PCC in MCI subjects comparing to controls.

Regions NC vs. MCI	BA	Cluster size	Coordinates (MNI)			T-score
			x	y	z	
Lt. Inferior Temporal gyrus	20	30	−63	−21	−21	6.25
Lt. Middle Temporal Gyrus	21	30	−66	−12	−9	3.90
Lt. Middle Temporal Gyrus	21	15	−54	0	−30	3.51
Lt. Superior Temporal Gyrus	39	33	−60	−60	21	3.28
Lt. Inferior Temporal Gyrus	20	15	−60	−6	−27	3.00
Lt. Superior Frontal Gyrus	6	12	−12	15	63	4.63
Rt. Superior Frontal Gyrus	9	34	27	45	36	4.51
Lt. Anterior Cingulate Cortex	32	27	−12	42	6	4.19
Lt. Medial Prefrontal Cortex	10	30	−18	45	−3	3.58
Rt. Medial Prefrontal Cortex	10	19	6	51	15	3.52
Lt. Medial Prefrontal Cortex	10	12	−6	60	15	3.51
Rt. Medial Prefrontal Cortex	10	9	6	57	15	3.05
Rt. Middle Frontal Gyrus	8	34	27	30	45	2.50
Lt. Precuneus	7	56	−3	−69	42	4.86
Rt. Posterior Cingulate Cortex	30	65	6	−48	18	4.35
Rt. Precuneus	7	65	3	−72	42	4.12
Lt. Posterior Cingulate Cortex	23	8	−3	−57	12	3.90
Lt. Precuneus	7	11	−3	−39	48	3.86
Lt. Angular Gyrus	39	9	−45	−72	36	3.29
Rt. Subthalamic Nucleus	–	8	9	−12	−3	4.31

## Results

### Demography and Neuropsychological Test

Demographic characteristics and neuropsychological scores were shown in Table 1a. There were no significant differences between the MCI group and control group in gender, age, and years of education, but the neuropsychological test such as MMSE and CVLT scores were significantly different (P<0.05) between the two groups.

Seven MCI patients were traced using the same criteria and methods. Demographic characteristics and neuropsychological scores were shown in Table 1b. The changes of neuropsychological scores suggested that there was a trend of cognitive decline over the period, although the difference was not very significant.

Nine controls were traced using the same criteria and methods. Demographic characteristics and neuropsychological scores were shown in Table 1c. There were no significant differences of neuropsychological scores between the two groups.

**Figure 2:**
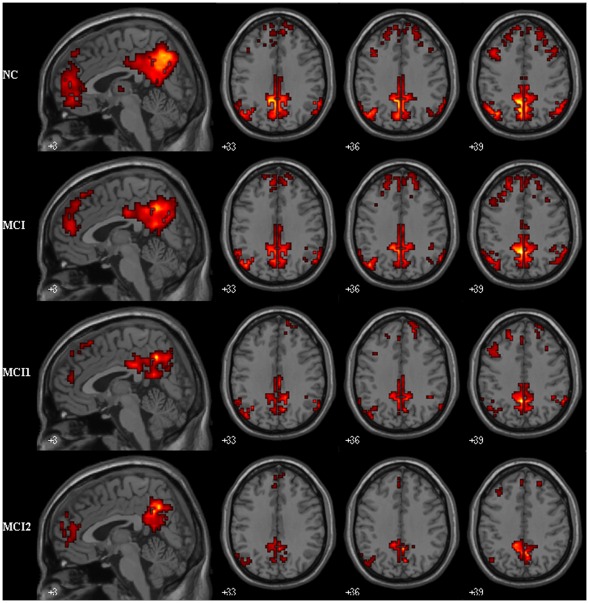
Within group maps of PCC connectivity in MCI and control group. Brain regions show significant connectivity to PCC within controls (14 subjects), MCI (14 patients), MCI1 (7 patients) and MCI2 (7 patients) group with P<0.01 and a minimum cluster size of 10 voxels. Left in picture is left in the brain.

**Figure 3:**
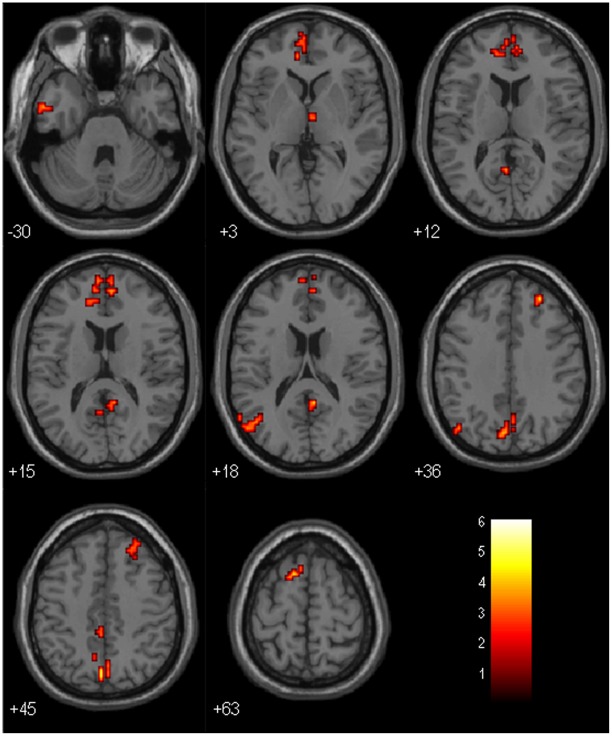
PCC connectivity between MCI and Control group (with GM correction). Brain regions showing decreased connectivity to PCC in MCI group comparing to control group. Left in picture is left in the brain. The color scale represents t values.

### VBM Analysis

Compared with the control group, MCI patients showed a broad area of significant gray matter loss in the temporal lobe, frontal lobe, parietal lobe, occipital lobe as well as subcortical regions. The detailed regions include middle temporal gyrus (MTG), inferior temporal gyrus (ITG), superior temporal gyrus (STG), fusiform gyrus (FG), parahippocampal gyrus (PHG), superior frontal gyrus(SFG), inferior frontal gyrus (IFG), rectal gyrus (RG), precuneus, inferior parietal lobule (IPL), superior parietal lobule (SPL), middle occipital gyrus (MOG), inferior occipital gyrus (IOG), lingual Gyrus (LG) and caudate (p<0.05, Table 2 and Fig. 1a).

**Table 6:** Regions showing decreased and increased connectivity to the PCC in MCI2 subjects comparing to MCI1 subjects.

Regions	BA	Cluster size	Coordinates(MNI)					T-score
			x		y		z	
**MCI1 vs. MCI2**								
Lt. Middle Frontal Gyrus	8	19	−36		21	51		6.98
Rt. Middle Frontal Gyrus	6	16	42		9	57		3.67
Lt. Middle Frontal Gyrus	6	41	−24		21	60		3.54
Lt. Superior Frontal Gyrus	8	41	−24		27	54		3.45
**MCI2 vs. MCI1**								
Lt. Medial Prefrontal Cortex	9	18	−3	48		27		7.14
Lt. Medial Prefrontal Cortex	10	15	−3	54		0		6.34
Rt. Anterior Cingulate Cortex	32	25	6	51		0		6.14

Compared with MCI1 patients, MCI2 patients showed further gray matter loss in several regions: temporal lobe, frontal lobe, parietal lobe, occipital lobe as well as subcortical regions. The detailed regions include PHG, FG, STG, insula, MFG, SFG, IFG, orbital gyrus, precentral gyrus, IPL, paracentral lobule, postcentral lobule, MOG, caudate, lentiform nucleus and subthalamic nucleus (p<0.05, Table 3 and Fig. 1b).

Compared with control1 subjects, control2 subjects showed further gray matter loss in several regions: temporal lobe, frontal lobe, parietal lobe as well as subcortical regions. The detailed regions include MTG, MFG, precuneus, IPL, Supramarginal Gyrus (SMG) and caudate (p<0.05, Table 4).

**Figure 4:**
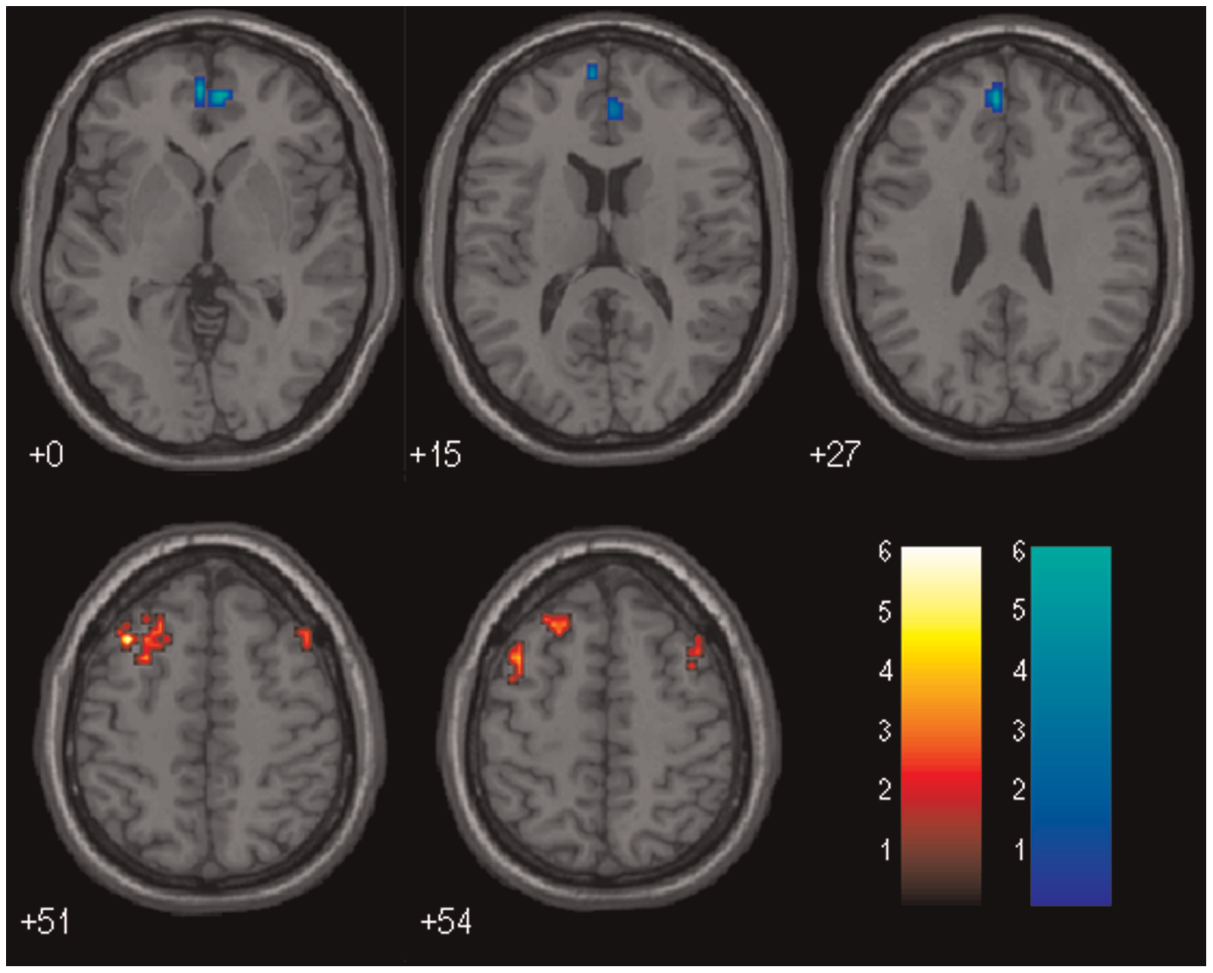
PCC connectivity between MCI2 and MCI1 group (with GM correction). Brain regions showing decreased and increased connectivity to PCC in MCI2 group comparing to MCI1 group: The above is MCI2>MCI1, the below is MCI1>MCI2. Left in picture is left in the brain. The color scale represents t values. Warm color represents decreased connectivity, while cool color represents increased connectivity.

**Figure 5:**
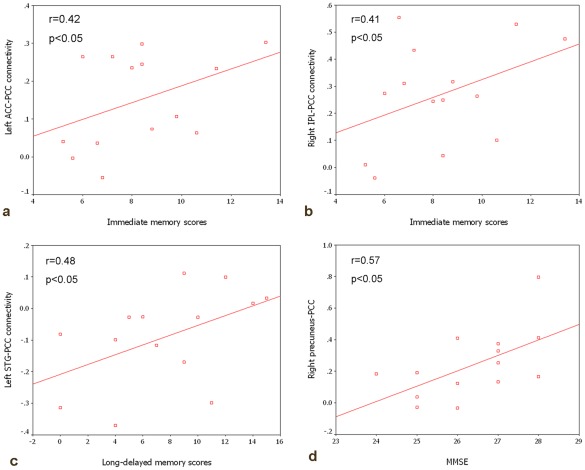
Correlation between PCC connectivity and neuropsychological scores in MCI group. a) left ACC-PCC connectivity and immediate memory scores (r = 0.42, p<0.05); b) right IPL-PCC connectivity and immediate memory scores (r = 0.41, p<0.05); c) left STG-PCC connectivity and long delayed memory scores (r = 0.48, p<0.05); d) right precuneus-PCC connectivity and MMSE (r = 0.57, p<0.05).

### PCC Connectivity: within-group Analyses

In both healthy controls and MCI patients, PCC showed strong connectivity to a number of brain regions. By visual inspection, these regions were mainly within DMN, such as the MPFC, PCC/PCu and so on (Fig. 2).

### PCC Connectivity between MCI Group and Control Group: Comparison after Gray Matter Correction

When comparing MCI group and control group, several regions showed significantly decreased connectivity to PCC in MCI group. These regions were ITG, MTG, STG, MPFC, SFG, MFG, anterior cingulate cortex (ACC), precuneus, PCC, angular gyrus (AG) and thalamus. Most of these regions are within DMN. There were no regions showing increased connectivity to PCC in MCI group. Results of the between-group comparison after gray matter correction were shown in Table 5 and Fig. 3.

### PCC Connectivity between MCI1 Group and MCI2 Group: Comparison after Gray Matter Correction

Seven MCI patients underwent resting-state fMRI examination 3 years later. The acquired fMRI data was analyzed with the same methods. PCC connectivity was compared between their initial stage and their stage 3 years later.

Comparing PCC connectivity between MCI1 group and MCI2 group, the region of SFG and MFG presented further decreased connectivity to PCC in MCI2 group. While, the MPFC and ACC showed a significantly increased connectivity to PCC in MCI2 group (Details see Table 6 and Fig. 4).

### PCC Connectivity between Control1 Group and Control2 Group: Comparison after Gray Matter Correction

Nine controls underwent resting-state fMRI examination 3 years later. There were no regions showing decreased or increased connectivity to the PCC in control2 subjects comparing to control1 subjects.

### Correlation between PCC Connectivity and Neuropsychological Scores in MCI Group

We studied the correlation between strength of PCC connectivity and neuropsychological data to explore whether changes in PCC connectivity could reflect the cognitive decline in MCI. Immediate memory scores were significantly and positively related to the left ACC-PCC connectivity and right IPL-PCC connectivity. Long–delayed memory scores were significantly and positively related to the left STG-PCC connectivity. In addition, MMSE scores were significantly and positively related to right precuneus-PCC connectivity (Details see Fig. 5a, b, c, d).

## Discussion

In the current study, three main findings are generated: first, multiple regions are functionally correlated with PCC and altered in MCI. Most of these regions are involved in the DMN; second, the PCC connectivity in MCI group showed some changes 3 years later; third, the strength of the PCC functional connectivity is independent of the gray matter atrophy of the MCI patients, and it is closely correlated to some neuropsychological scores.

Also in this study, the frontal lobe (SFG/MFG/MPFC/ACC), the parietal lobe (PCC/precuneus/AG), the temporal lobe (STG/ITG/MTG), and thalamus appear to have disrupted connectivity to PCC in MCI. Most of these regions are among the DMN which is comprised of PCC/precuneus, MPFC/ACC, IPL, lateral temporal cortex (LTC) and so on [7], [26]–[27]. Connectivity of the DMN plays an important role in activity of human cognition and memory processing. There is decreased connectivity in DMN in AD patients, which has already been confirmed by various studies [4], [6], [7], [28]–[29].Such network might be affected by the amyloid deposition, atrophy as well as functional disruption in parietotemporal regions [30]–[32]. In the previous studies, there are some controversial in the changes of the DMN in MCI patients. Some studies showed the selective disruption of the DMN in MCI patients [9]–[11], while another study found no change of DMN in MCI patients [12]. In the present study, the decreased connectivity between PCC and the frontal, parietal, temporal lobe suggests the memory impairment and cognitive decline in the MCI patients. This result is consistent with many previous studies in MCI.

We also observed that the subcortical region, such as the thalamus, presented decreased connectivity to PCC. In the previous studies, the thalamus is considered a vital region which seems to integrate neural activity from widespread neocortical inputs and outputs [33]. The thalamus is believed to modulate and facilitate communication in all areas of the cerebral cortex. The disruption between the thalamus and PCC in MCI suggests the cognitive decline. In our study, we selected thalamus as ROI to explore the functional connectivity in MCI and found the similar results [34].

When comparing the PCC connectivity between the initial stage in MCI1 and those 3 years later in MCI2, the regions of SFG and MFG presented further decreased connectivity in the longitudinal study of MCI patients. We attributed it to the more severe pathology of the MCI patients. On the other hand, we noticed that the MPFC and ACC connectivity to PCC showed decrease in the initial stage and increase 3 years later. We speculated that our finding might be explained as follows: early in the course of MCI when memory deficits were less prominent, there might be decreased PCC connectivity with other regions including the MPFC and ACC, later in the course of MCI, with the further decline of the cognition, the PCC connectivity with the MPFC and ACC gradually converted to increase, possibly representing inefficient compensatory activity. It suggests that MCI patients may apply the enhanced anterior-posterior connectivity to compensate for the disruption of the DMN in the advanced stage of the disease. In the previous studies, many researchers found the enhanced connectivity in the frontal regions in AD patients [4], [18], [28], [35]–[37]. However, there is also discrepancy in the connectivity of frontal regions in MCI patients. Some studies revealed the increased connectivity of frontal regions in these patients [8], [9], [11], while another study demonstrated the decreased functional connectivity in the frontal regions as well as PCC and so on [10]. In addition, another recent study did not show alteration in the connectivity of frontal regions in MCI patients at all [28]. We attributed it to different samples and analyzed methods.

It should be noted that we also examined the gray matter atrophy in MCI patients using VBM analysis. We found that there were diffuse gray matter losses in several regions in MCI patients. With the progress of the disease, the gray matter atrophy became more severe, which may impact on the analysis of the resting state data. This seems even more relevant in longitudinal comparisons. So we took global measure of brain atrophy as a covariate to analyze the resting state data. After controlling for gray matter volumes, there was significant functional connectivity disruption in MCI patients comparing with the control group. This might reveal that the functional connectivity alteration was independent of the gray matter atrophy in MCI patients. In other words, it supported the idea that the observed changes in functional connectivity were not the expression of brain atrophy.

We also explored the correlations between the resting-state PCC connectivity and cognitive performances, for example, the strength of the right precuenus-PCC connectivity showed significant positive correlation with MMSE scores in MCI patients. This might imply that the right precuenus-PCC decreased connectivity may contribute to the cognitive decline in the MCI patients. The more decrease of the connectivity strength, the more decline of the cognitive performance. The correlation between the PCC connectivity and the immediate and long-delayed memory scores also presented the same situation.

There are also several limitations in our study. First, although we kept the scanner room dim during the scanning and instructed the subjects not to think of anything, the subjects may still think something subconsciously that we could not control. Second, we couldn’t exclude the interference of some potential confounding factors, such as respiratory and cardiac cycle artifacts, slow sampling rates. It is known that slow sampling rates (as in this study TR = 2 s), noise from the cardiac and respiratory cycle can alias into the resting-state low frequency ranges [22]. Finally, a large number of samples may be statistically helpful.

In summary, the current study tried to explore the baseline and longitudinal patterns of the PCC connectivity using the resting-state fMRI, and found that impairment and compensation coexist in the disease progress of MCI patients. We also found that PCC functional connectivity was independent of the gray matter atrophy. Finally, we found there were close relationships between PCC connectivity changes and cognitive performances.
